# Editorial: Identification and characterization of contrasting genotypes/cultivars to discover novel players in crop responses to abiotic/biotic stresses, volume II

**DOI:** 10.3389/fpls.2022.1105598

**Published:** 2022-12-20

**Authors:** Raul A. Sperotto, Felipe K. Ricachenevsky, Elizabeth R. Waters, Guihua Bai, Magdalena Arasimowicz-Jelonek

**Affiliations:** ^1^ Graduate Program in Biotechnology, Life Sciences Area, University of Taquari Valley - Univates, Lajeado, Brazil; ^2^ Graduate Program in Plant Physiology, Federal University of Pelotas, Pelotas, Brazil; ^3^ Graduate Program in Cell and Molecular Biology, Botany Department, Federal University of Rio Grande do Sul, Porto Alegre, Brazil; ^4^ Department of Biology, San Diego State University, San Diego, CA, United States; ^5^ USDA-ARS Hard Winter Wheat Genetics Research Unit, Manhattan, KS, United States; ^6^ Department of Plant Ecophysiology, Faculty of Biology, Adam Mickiewicz University, Poznań, Poland

**Keywords:** biotic factors, drought, heat, nutrition, salinity, shade

Securing global food supply in an increasingly volatile climate and rapidly growing population is one of the greatest challenges facing humanity in the current century (Yan et al., 2022). Increased frequency and intensity of abiotic and biotic stress will demand a sustainable increase in food productivity with more efficient and diversified agricultural management (Farooq et al., 2022). One recurrent strategy used by plant scientists is to identify and characterize plant genotypes/cultivars with contrasting responses to these stressful conditions (Sperotto et al., 2021; Habibpourmehraban et al., 2022; Somaddar et al., 2022; Yu et al., 2022; Zhao et al., 2022), allowing the identification of molecular, biochemical, and physiological mechanisms involved in crop response and stress tolerance. This improved knowledge can be used to boost crop growth and productivity even under non-optimal conditions (Zhang H. et al., 2022). Therefore, this Research Topic presents an update on the advances to understanding plant responses to stressful conditions and provides an overview of different approaches used for improving crop tolerance/resistance. Here we highlight the major points arising from these reports.

## Heat-related

Global warming due to climate change affects plant growth and development throughout its life cycle. In addition, the increasing occurrence of heat waves is drastically reducing the global crop yield (Haider et al., 2022). Due to accelerating climate change, there is an urgent need across all crop species to identify heat tolerant varieties and to understand the genetic and physiological traits that provide resilience to heat stress. Tomás et al. evaluated whole transcriptomic profiles of wheat developing grains of commercial genotypes and landraces submitted to heatwave-like treatments during grain filling. Landraces showed more differentially expressed genes and presented more similar responses than commercial genotypes, showing that landraces are more affected by the high temperature treatment. Only six upregulated genes were detected in all four evaluated genotypes: three small heat shock proteins HSP20, one adenylate kinase (essential for cellular homeostasis), a BAG domain protein (responsible for the modulation of chaperones activity), and a ferritin (recently related with increased tolerance to several abiotic stresses in Arabidopsis - Zang et al., 2017). Additionaly, the commercial variety Bancal seems to be a promising genotype to cope with high temperatures.

Li et al. reported on a detailed study of two alfalfa (*Medicago sativa*) varieties that differ in their ability to tolerate heat stress. Plants were exposed to continuously rising temperatures up to a maximum of 43°C. It was reported that the heat-tolerant variety (MS30) was able to maintain higher chlorophyll fluorescence rates and had lower rates of electrolyte leakage during heat stress. In addition, the antioxidant defense system was also higher in MS30 compared to the heat-sensitive (MS37) variety. Proteomic analysis revealed that in both varieties photosynthesis-related genes were differentially regulated, as were the heat shock proteins. It is interesting that the sensitive variety MS37 had a higher number of differentially regulated proteins, suggesting that the tolerant MS30 has a higher ability to buffer plant metabolism during heat stress.

Abro et al. reported on a study of fifty-eight cotton genotypes. These authors utilized a heat-susceptibility index (HSI) that evaluates yield during control conditions compared to yield under heat stress. In addition, they examined electrolyte leakage as well as stomata and trichome size. Seventeen of the varieties examined were found to be heat-tolerant based on HSI and low electrolyte leakage. There was no clear relationship between the size of trichomes and heat tolerance, but there was a clear relationship between stomata size and heat tolerance. This study indicates that stomata size and electrolyte leakage will be useful in future studies of cotton heat-tolerance.

Devi et al. examined 39 field-grown genotypes of *Cicer arietinum* (chickpea) for evidence of genetic variation in responses to heat stress. From this original analysis, they identified ten heat-sensitive and ten heat-tolerant varieties for more detailed characterization. Heat-tolerant varieties displayed higher chlorophyll content, chlorophyll fluorescence, lower electrolyte leakage, higher stomatal conductance, higher pollen germination and viability. The heat-tolerant chickpea varieties identified in this study can now be utilized for crop improvement to generate varieties that will maintain higher yield as the climate warms and heat stress becomes more prevalent.

## Drought-related

Among the abiotic stresses that affect plants, drought is one of the main factors which reduces growth and yield, with an estimated third of the total cultivated area affected by water deficit. Therefore, we need to improve our knowledge of the molecular mechanisms underlying drought tolerance in order to develop new drought tolerant genotypes (Fadoul et al., 2021). In light of ever increasing climate change and the new agricultural challenges this brings, Álvarez-Maldini et al. emphasized the importance of identifying and selecting anisohydric woody plants that are able to withstand cavitation better than isohydric plants. Based on pot desiccation experiment, the authors described anisohydric and isohydric behavior of the selected almond (*Prunus dulcis*) cultivars and indicated that anisohydric ones, Soleta and Isabelona, are characterized by maximum stomatal conductance, lower water potentials for stomatal closure and turgor loss, and lower vulnerability to xylem cavitation. They found that almond can avoid cavitation by closing stomata during the early stages of drought. In addition, they reported that root system architecture (RSA) plays a vital role in plant productivity under water stress.

Abdirad et al. compared the transcriptomic responses of root tips to water stress between a shallow-rooting drought-susceptible rice cultivar IR64 and a drought-tolerant and deep-rooting cultivar Azucena. They found that Azucena avoided water stress through enhancing growth and exploration of roots to access water from deep soil, whereas IR64 relied on cell insulation to maintain water and antioxidant system to withstand stress. Many putative candidate genes were identified as associated with RSA and drought tolerance, some of which may have the potential to be used to enhance drought adaptation in rice.

## Shade-related

Depending on the strategy adopted to cope with vegetative shade, plants can be classified into two groups: shade avoiding or shade-tolerant species. Species typically found in forest understories exhibit shade tolerance responses, which allows them to maintain adequate growth under shade conditions (Xu et al., 2021). Cheng et al. presented new aspects of the lodging resistance mechanism of soybean in the strip intercropping system, which is especially important in areas with low solar radiation. Using a shade-tolerant soybean in the strip intercropping system could reduce yield loss by regulating its spatial canopy structure and stem characteristics. In the field, Tianlong No. 1 cultivar showed increased light energy capture and photosynthesis that favor morphological and physiological development of the stem. Parameters characterizing the canopy spatial structure such as high transmission coefficient (TC) and low leaf area index (LAI) and mean leaf angle (MLA) could be used in the future as indicators for screening soybean cultivars with shade and lodging tolerance.

## Salt-related

Salt stress is a widespread problem in both agriculture and natural environments. It is predicted that 50% of today’s arable land will be lost to salinization in the future (Goyal et al., 2016), making the identification of salt tolerant species and genotypes and the understanding of salt-tolerance mechanisms a priority. Currently, breeding salinity-tolerant cultivars is the most promising solution for salinity tolerance in high-yielding crops (Zhang J. et al., 2022). Wheat is one of the most important crops worldwide, but it is sensitive to salt stress. Another grass from the Triticeae tribe, *Thinopyrum elongatum*, is a halophyte that can generate hybrids when crossed with the common wheat variety ‘Chinese spring’. Peng et al. performed biochemical, physiological, and molecular comparisons between common wheat and wheat/*T. elongatum* hybrids, named *Tritipyrum*, which are highly salt-tolerant, exposed to NaCl. Tolerance was associated with increased antioxidant enzyme activity, proline and soluble sugar levels, and lower detrimental effects on photosynthesis in *Tritipyrum* compared to wheat. Transcriptomic analyses suggested a quantitative effect, rather than qualitative, of known salt tolerance mechanisms. This work provides interesting new information in an important breeding material for generating salt-tolerant wheat genotypes.

Hou et al. demonstrated that wild soybean (*Glycine soja*) is more salt-tolerant than cultivated soybean, and associated the natural sequence variation in dehydration responsive element-binding (DREB) family transcription factor genes (*DREB3a* and *DREB3b*) with salt tolerance in soybean. Soybean plants carrying the wild soybean *DREB3b* allele (*DREB3b39Del*) were more salt-tolerant than those containing the reference genome allele (*DREB3bRef*). Domestication of cultivated soybean that lost the *DREB3b39Del* allele may be associated with a reduction in salt tolerance.

Zhang et al. treated two sorghum species, *LRNK1* (salt-tolerant (ST)) and *LR2381* (salt-sensitive (SS)) with 180 mM NaCl salt solution, and combined transcriptomic, metabolomic, and physiological analyses to elucidate key biological pathways in sorghum response to salinity stress. Under salt stress, the ST species presented higher plant height, leaf area, and chlorophyll contents than the SS one, besides accumulating higher levels of salicylic acid and betaine. Additionally, the expression of *SbNPR1*, a salicylic acid receptor, was positively correlated with salt tolerance. This study will play an important role in identifying salt-tolerant varieties that can be used in sorghum breeding programs.

Zhang et al. compared three rice genotypes under different NaCl concentrations. They showed that panicle number, grain number per panicle, and 1,000 grains weight were reduced at high NaCl concentration. However, salt-sensitive genotypes in fact increased yield components when exposed to low NaCl concentration, whereas tolerant genotypes did not. A similar trend was observed in grain quality, in which salt tended to decrease product quality, but low NaCl level could improve important properties for the consumers. The study should be followed up by more extensive works with other rice varieties, which could lead to increased rice production in salt-containing areas such as mudflats.

## Nutrient-related

Plants require both macro and micro nutirents to complete their life cycles. Plant growth and productivity can be severely affected by disbalanced nutrient availability, and different adaptations have enabled plants to cope with inappropriate nutrient levels (Pita-Barbosa et al., 2019; Islam et al., 2022). Adequate plant nutrition is a key problem that needs to be tackled to maintain our food security quality in the future when the human population will reach more than 9 billion people. Yam (*Dioscorea* spp.) is a key crop, especially in West Africa. Using six white Guinea yam (*Dioscorea rotundata*) genotypes, Matsumoto et al. tested how NPK (Nitrogen, Phosphorus, Potassium) fertilization affects biomass production and nutrient use efficiency in field trials. The varieties responded differently to fertilization with NPK. The authors identified the varieties that increase tuber dry matter, nutrient efficiency, and recovery, and those susceptible to variation in soil fertility. This work will help farmers identify genotypes more suitable to each condition, especially those that can produce quality biomass when soil fertility is low and nutrient input is not adequate.

## Biotic stress-related

Biotic stresses are crucial constraints to the productivity of crop plants, especially under climate change conditions (Parihar et al., 2022), and lead to more than 20% crop yield losses (Savignac et al., 2022). Galewski et al. identified specific genomic features associated with seedling rhizoctonia resistance by whole genome sequencing of selected and unselected bulk derived from a synthetic outcrossing sugar beet population and the genome-wide variation resulting from the selection. An increased level of allele fixation in the resistant bulk indicated that a greater selection pressure was applied. Using population genetics and statistics approaches, and the EL10.2 physical map, a list of enriched candidate genes was identified with the functions in cell wall metabolism and plant disease resistance, including pathogen perception, signal transduction, and pathogen response.

The brown planthopper (*Nilaparvata lugens* Stål, BPH) belongs to the most devastating pest of rice that causes significant yield loss. By the application of bulked segregant RNA sequencing, Guan et al. identified candidate genes involved in BPH virulence. Comparison of virulent and avirulent BPH extreme bulks after feeding on YHY15 rice plants carrying the *Bph15* resistance gene allowed to identify 751 differentially expressed genes (DEGs). Functional analysis of 418 DEGs with upregulated expression in the avirulent insects revealed that BPH induces carbohydrate, amino acid, and nucleotide metabolism, endocrine system, and signal transduction pathways in response to feeding on YHY15 rice. Moreover, authors identified 24 polymorphic SNPs related to BPH virulence and 21 genes potentially engaged in virulence mechanisms.

## Final comment

In summary, the studies presented here documents how contrasting genotypes differently cope with abiotic and biotic stress conditions. The identification of these tolerance/resistance mechanisms can be used in the future to enhance plant abilities to respond to unfavorable environmental conditions.

## Author contributions

All authors listed have made a substantial, direct, and intellectual contribution to the work and approved it for publication.
